# Psychedelics and Suicide-Related Outcomes: A Systematic Review

**DOI:** 10.3390/jcm14051416

**Published:** 2025-02-20

**Authors:** Shakila Meshkat, Taha Malik, Richard Zeifman, Jennifer Swainson, Yanbo Zhang, Lisa Burback, Olga Winkler, Andrew J. Greenshaw, Amy Claire Reichelt, Eric Vermetten, David Erritzoe, Manish K. Jha, Walter Dunn, Rakesh Jetly, Muhammad Ishrat Husain, Venkat Bhat

**Affiliations:** 1 Interventional Psychiatry Program, St. Michael’s Hospital, Toronto, ON M5B 1M8, Canada; shakila.meshkat@unityhealth.to (S.M.);; 2 NYU Center for Psychedelic Medicine, NYU Grossman School of Medicine, New York, NY 10016, USA; 3 Center for Psychedelic Research, Department of Brain Sciences, Imperial College London, London SW7 2AZ, UK; 4 Department of Psychiatry, University of Alberta, Edmonton, AB T6G 2R3, Canada; 5 Neuroscience and Mental Health Institute (NMHI), University of Alberta, Edmonton, AB T6G 2R3, Canada; 6 Department of Physiology and Pharmacology, Western University, London, ON N6A 5C1, Canada; 7 School of Biomedicine, University of Adelaide, Adelaide, SA 5005, Australia; 8 Department of Psychiatry, Leiden University Medical Center, 2333 ZA Leiden, The Netherlands; 9 Department of Psychiatry, UT Southwestern, Dallas, TX 75247, USA; 10 The Department of Psychiatry, University of California, Los Angeles, CA 90095, USA; 11 The Institute of Mental Health Research, University of Ottawa, Ottawa, ON K1Z 7K4, Canada; 12 Campbell Family Mental Health Research Institute, Centre for Addiction and Mental Health, Toronto, ON M6J 1H4, Canada; 13 Department of Psychiatry, University of Toronto, Toronto, ON M5S 3E5, Canada

**Keywords:** suicide, suicidal behavior, psychedelics, psilocybin, MDMA, psychiatric disorders, mood disorders, mental health

## Abstract

**Background/Objectives**: Suicide accounts for 1.4% of global deaths, and the slow-acting nature of traditional treatments for suicide risk underscores the need for alternatives. Psychedelic therapies may rapidly reduce suicide risk. This systematic review evaluates impact of psychedelic therapies on suicide-related outcomes. **Methods:** A systematic search of MEDLINE, Embase, PsycINFO, and ClinicalTrials.gov was conducted up to November 2024. **Results**: Four randomized controlled trials (RCTs) evaluated suicidality as a secondary outcome or safety measure, showing significant reductions in suicidal ideation with psilocybin (three studies) and MDMA-assisted therapy (MDMA-AT; one study). Effect sizes, measured by Cohen’s d, ranged from =0.52 to 1.25 (*p* = 0.01 to 0.005), with no safety issues reported. Five additional RCTs assessed suicidality as a safety measure, showing reductions in suicidal ideation with psilocybin (two studies) and MDMA-AT (three studies; *p* = 0.02 to 0.04). Among 24 non-randomized and cross-sectional studies, results were mixed. Psilocybin (three studies) reduced suicidal ideation, with odds ratios (OR) of 0.40–0.75. MDMA-AT (five studies in PTSD patients) had a pooled effect size of *d* = 0.61 (95% CI: 0.32–0.89). LSD (six studies) showed increased odds of suicidality, with odds ratios ranging from 1.15 to 2.08. Studies involving DMT (two studies) and multiple psychedelics (three studies) showed mixed results, with DMT studies not showing significant effects on suicidality and studies involving multiple psychedelics showing varying outcomes, some reporting reductions in suicidal ideation and others showing no significant change. **Conclusions**: The effect of psychedelic therapies on suicide-related outcomes remains inconclusive, highlighting the need for further trials to clarify safety and therapeutic mechanisms.

## 1. Introduction

Suicidal thoughts and behaviors, encompassing completed suicide, suicide attempts, and suicidal ideation, represent a major public health challenge and a significant contributor to global mortality [1]. Death by suicide accounts for approximately 1.4% of all deaths worldwide and ranks as the 10th leading cause of death in the United States [2]. Suicidal behavior arises from a complex interplay of factors, broadly categorized into predisposing elements and immediate stressors or triggers [3]. Mental disorders play a predominant role, with over 90% of individuals who die by suicide meeting the criteria for a psychiatric illness as outlined in the Diagnostic and Statistical Manual of Mental Disorders, Fourth Edition (DSM-IV) [4]. Among psychiatric conditions, mood disorders—primarily major depressive disorder (MDD) and bipolar disorder (BD)—are implicated in approximately 60% of completed suicides [5]. Notably, trauma burden and posttraumatic stress disorder (PTSD) significantly increase the risk of suicidal ideation and behaviors, further compounding the challenge of prevention and treatment [6]. Additional influences include the availability of lethal means, substance use (alcohol and drugs), access to mental health care, cultural and personal attitudes toward suicide, help-seeking behaviors, physical health conditions, marital status, age, and gender [3,7]. Current biological approaches to reduce suicidality remain limited in their effectiveness. Traditional treatments, such as antidepressants, cognitive behavioral therapy (CBT), and electroconvulsive therapy (ECT), have shown to reduce suicidal thoughts and behaviors in individuals with depression [8,9,10]. Additionally, pharmacotherapies such as clozapine and lithium, recognized for their anti-suicidal properties, also played a significant role in managing suicidality [11]. However, these interventions typically require several weeks to take effect, leaving many patients at significant risk of suicide during this period. Recent developments in rapid-acting antidepressants (RAADs), such as ketamine and its FDA-approved derivative esketamine (Spravato), represent promising advances in the treatment of acute suicidal ideation and behaviors, particularly in individuals with depression [12]. While these interventions can reduce symptoms within hours, their effects are limited to this population and do not address suicidality in a broader sense. Additionally, the short duration of effect following a single treatment and the unknown durability of their anti-suicidal properties with repeated administrations pose challenges to their clinical application [13]. Therefore, there is an urgent need for novel efficacious treatments to address this critical issue.

Serotonergic psychedelics, commonly known as “classic” psychedelics, include a range of substances such as psilocybin, dimethyltryptamine (DMT), ayahuasca, mescaline, and lysergic acid diethylamide (LSD) [14]. In contrast, 3,4-methylenedioxymethamphetamine (MDMA) is a distinct psychoactive compound considered an empathogen or entactogen that has also garnered attention for its therapeutic potential [14]. Classic psychedelics are naturally occurring, plant-derived or synthesized psychoactive substances that primarily function as serotonin 2A receptor agonists [15]. These compounds have the potential to induce profound experiences, which, in some cases, can enhance an individual’s perceived quality of life [15]. Similarly, MDMA (3,4-methylenedioxymethamphetamine), though not a classic psychedelic, acts primarily as a serotonin-releasing agent and promotes prosocial effects, emotional openness, and enhanced therapeutic processing, which may also contribute to improvements in perceived quality of life [16,17]. Psychedelics, administered with psychological support, have been investigated in numerous randomized clinical trials (RCTs) for a variety of psychiatric conditions, including MDD, BD, treatment-resistant depression (TRD), substance use disorders (SUDs), and PTSD [18,19,20,21,22]. Psychedelic therapies may also have potential benefits in reducing suicidality due to their ability to enhance emotional processing, facilitate personal insights, and promote a sense of interconnectedness, which can improve mental health and reduce self-destructive thoughts [23]. However, psychedelic use is also linked to adverse events, including heightened anxiety [24], and there have been reports suggesting an increase in suicidal ideations and behaviors in some individuals following LSD use [25].

Despite growing research on psychedelic therapies for psychiatric disorders such as depression, anxiety, substance use disorders, and PTSD, their effects on suicidality remain underexplored. Notably, many clinical trials on psychedelic therapies exclude participants with suicidal ideation, often assessed via the clinician-rated Columbia–Suicide Severity Rating Scale (C-SSRS), or those with borderline personality disorder (BPD)—a population at elevated risk for suicide and comprising a significant portion of psychiatric patients. Given the urgent need for treatments targeting individuals with acute and severe psychiatric symptoms, identifying therapies that can address suicidality in these high-risk groups is of profound importance. A systematic review is needed to evaluate the impact of psychedelic therapies on suicide risk, clarify their safety and therapeutic mechanisms, and guide future research and clinical practice. Such an assessment is crucial for understanding how psychedelic therapies can be safely integrated into treatment for individuals at high suicide risk. Therefore, in this systematic review, we aim to evaluate the effect of psychedelic therapies on suicidal-related outcomes.

## 2. Methods

This systematic review followed the Preferred Reporting Items for Systematic reviews and Meta-Analyses guidelines [26] and was registered on PROSPERO (registration ID: CRD42024611536).

### 2.1. Search Strategy

We conducted a comprehensive search in three databases (MEDLINE, Embase, and PsycINFO) via OVID, covering all records from inception through November 2024. We also searched Google Scholar and references of relevant studies. The following keywords were used: psilocybin or psilocibin or psilocybine or silocybin or psiloc* or shrooms or magic mushrooms or mushies or psilocybin-assisted therapy or PAP or psychedelics or MDMA or 3,4-methylenedioxymethamphetamine or ecstasy or molly or ayahuasca or LSD or lysergic acid diethylamide or DMT or dimethyltryptamine or mescaline or peyote or ibogaine or iboga or 5-MeO-DMT or salvinorin A or Salvia or bufotenin or 5-HO-DMT AND suicide or suicidal ideation or suicidality or suicidal thoughts or suicidal behavior or suicide attempt. No language or publication date restrictions were applied. Additionally, a search of registered clinical trials was conducted on ClinicalTrials.gov on 21 November 2024, using the same psychedelic agents paired with suicide-related terms.

### 2.2. Eligibility Criteria

Two reviewers (SM, TM) independently reviewed the title/abstract and full text of studies based on eligibility criteria. The screening process was conducted in Covidence (https://www.covidence.org/ (accessed on 1 November 2024). Conflicts between reviewers were resolved through discussion, and if disagreements persisted, a third reviewer was consulted to reach a consensus (VB). Studies were included if they met the following criteria: (1) original research of any study design; (2) evaluation of the effect or association of psychedelics or hallucinogens with suicide-related outcomes; (3) published in English. Secondary analyses were included only if their primary focus was on the effects of psychedelics on suicidal-related outcomes. Qualitative studies were also considered. Excluded studies were case reports, non-human studies, systematic reviews, narrative reviews, umbrella reviews, meta-analyses, letters, editorials, posters, conference abstracts, and studies not published in English. We chose to exclude systematic reviews and meta-analyses to ensure that our findings are based on primary data rather than synthesized evidence, thereby avoiding potential duplication of results. Regarding the exclusion of non-English studies, this decision was made to ensure consistency in data extraction and interpretation, as translations may introduce variability or misinterpretation of critical findings.

### 2.3. Data Extraction

Two reviewers (SM, TM) independently reviewed the full texts of eligible studies and extracted the following variables: author, year of publication, country, study design, participants, intervention, psychotherapy principle, outcome measure, results, and conclusion. Similar to the published studies, the following variables were extracted from the registered clinical trials: study characteristic, intervention, outcome measure, country, diagnosis, and estimated completion date. Additional extracted variables were the ClinicalTrials.gov identifier (i.e., an 11-digit alphanumeric identifier), trial status (i.e., not yet recruiting, recruiting, active, completed, etc.), estimated completion date, and projected sample size.

### 2.4. Quality Assessment

All studies included in this review were evaluated for quality by two independent assessors (SM, TM) using the JBI Critical Appraisal Tools Checklist for systematic reviews [27]. The specific checklists applied were tailored for RCTs and cohort studies. Lower-quality studies were not excluded; however, their methodological limitations were considered in the interpretation of findings. While a formal sensitivity analysis was not conducted, the influence of these studies was evaluated qualitatively, with particular attention to study limitations (Supplementary Materials Table S1).

## 3. Results

### 3.1. Search Results

**Figure 1:**
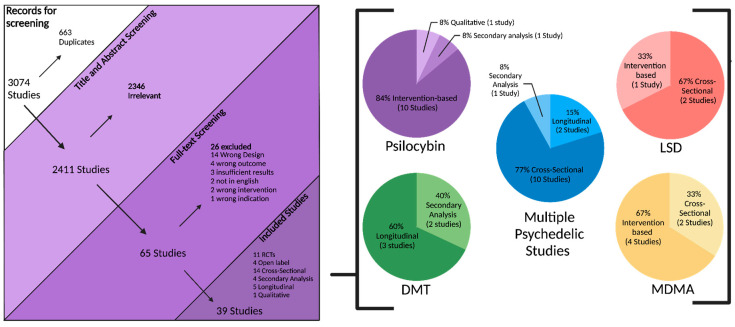
Study flow diagram.

**Figure 2:**
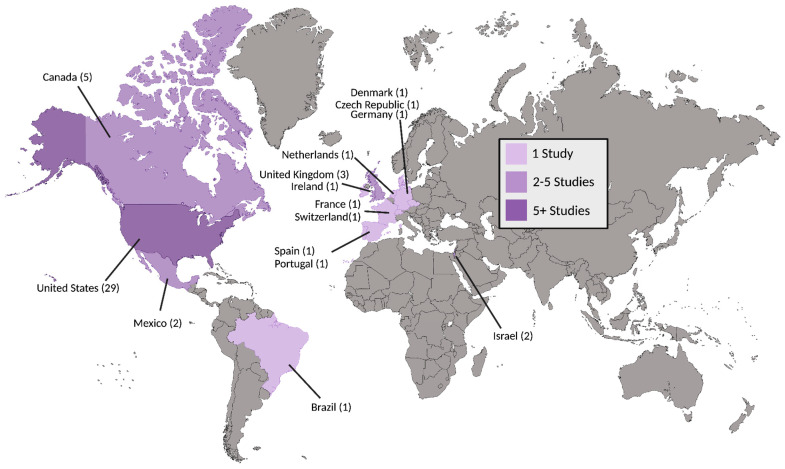
Countries of included studies. Number of studies for a given country are included in parentheses.

**Figure 3:**
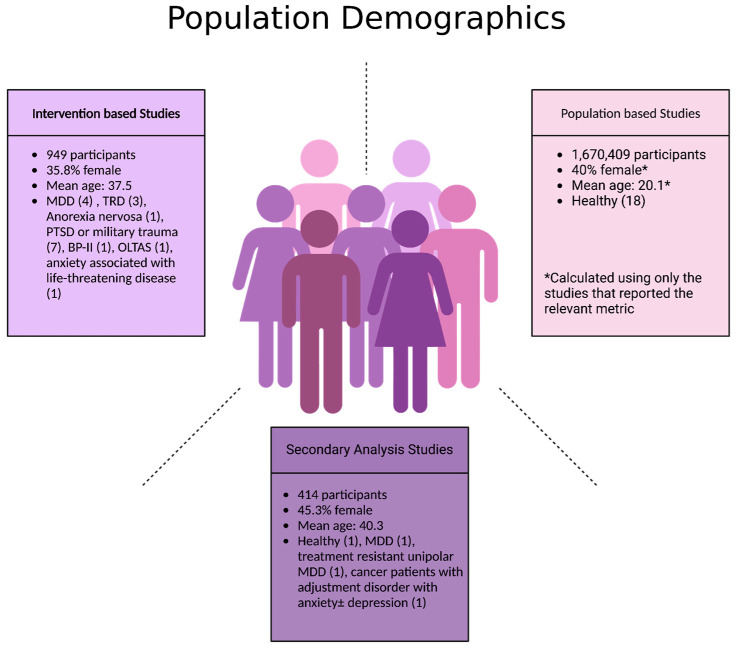
Population demographics of participants in included studies. Abbreviations: MDD: major depressive disorder; TRD: treatment-resistant depression; PTSD: post-traumatic stress disorder; BP-II: Bipolar II disorder; OLTAS: older long-term acquired immunodeficiency syndrome survivors.

**Figure 4:**
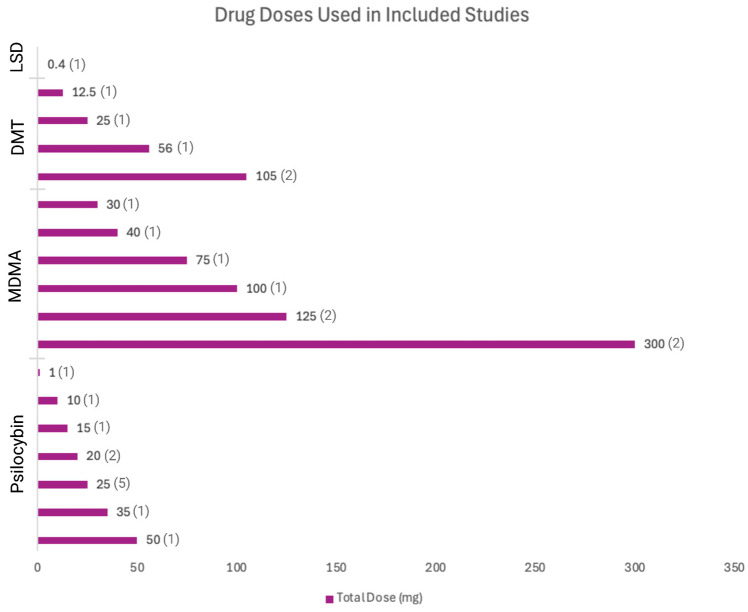
Drug doses used in included studies.

An electronic OVID database search resulted in a total of 3074 records. We removed duplicate articles (*n* = 663) and screened the title/abstract of the remaining records (*n* = 2411). A total of 2346 articles were excluded based on title/abstract. We reviewed the full text of the remaining papers (*n* = 65) in detail based on our inclusion criteria. Out of 65 records, 26 records were excluded: 14 studies had the wrong study design, 4 did not include suicidal-related outcomes, 3 had insufficient results, 2 had the wrong intervention, 2 were not in English, and 1 had the wrong indication. Finally, 39 articles involving 1,671,773 participants (949 from intervention-based studies, 1,670,409 from population-based studies, and 415 from secondary analysis studies) met the inclusion criteria and were included in this systematic review. We also found one ongoing trial on ClinicalTrials.gov (accessed on 21 November 2024). The study selection details are indicated in Figure 1. The characteristics of the included studies are listed in Table 1 and Table 2 and Figure 2, Figure 3 and Figure 4.

### 3.2. Registered Clinical Trial

We found one trial that had the identifier NCT05220410, which is currently recruiting participants. The estimated completion date for the study is April 2024, and it is being conducted in the United States. This is an interventional phase 2 trial with a single-group assignment and no masking. The projected sample size is 20 participants. The intervention involves administering 25 mg of psilocybin, and the primary outcome measure is the Columbia–Suicide Severity Rating Scale (C-SSRS). The trial focuses on individuals with TRD with chronic suicidal ideation.

### 3.3. Psilocybin

Six RCTs, four open-label trials, one qualitative cross-sectional study, and one secondary analysis investigated the effects of psilocybin on suicidal ideation and attempt.

#### 3.3.1. RCTs with Suicide as Safety or Secondary Outcome

##### MDD

Carharrt-Harris et al. (2021) examined the effects of psilocybin on suicidal thoughts through the Suicidal Ideation Attributes Scale (SIDAS; out of 50, 5 items out of 10) in MDD patients [28]. Their psilocybin group received two doses of 25 mg psilocybin 3 weeks apart (and daily placebo capsules), whereas their comparator group received two doses of 1 mg psilocybin with 6 weeks of oral escitalopram. All participants also received psychological support. The investigators reported a −2.0 change in the psilocybin group (95% CI = −4.3 to 0.0), a −0.8 change in the placebo group (95% CI = −3.4 to 2.0), and a between-group difference of −1.3 (95% CI = −6.5 to −0.3) from baseline to 6 weeks post dosing, but these were not corrected for multiple comparisons [28]. Davis et al. (2021) administered two doses of psilocybin (20 mg/kg, followed by 30 mg/kg) with psychotherapy to patients with MDD [29]. Patients either received treatment directly after baseline screening (immediate group) or after 8 weeks (delayed group). As a secondary outcome, Davis et al. assessed patients at baseline and clinical endpoints (i.e., time of follow-up) with the clinician-administered C-SSRS intensity of ideation subscale (C-SSRS-ISS; 0 out of 5), and found non-significant reductions in suicidal ideation from baseline (immediate: 1.2, delayed: 1.3; *p* = ns) to 5 (immediate: 0.2, delayed: 0.6; *p* = ns) and 8 (immediate: 0.2, delayed: 0.4; *p* = ns) weeks out [29]. They state that in general, suicidal ideation was low at baseline and trended lower after treatment [29]. Importantly, as participants with medically significant attempts for suicide were excluded from this study, observed C-SSRS-IIS scores may be lower among this population than otherwise. Raison et al. (2023) also investigated MDD patients and conducted an RCT using a single 25 mg dose of psilocybin with psychotherapy [32]. In their safety assessment, they report that no patients showed signs of suicidal behavior at any timepoint during the trial, as measured through the clinician-administered C-SSRS-IIS or the Montgomery–Åsberg Depression Rating Scale-Suicidality Item (MADRS-SI; out of 6) [32]. However, one participant receiving psilocybin and five receiving placebo niacin had an increase in C-SSRS suicidal ideation score from baseline to the end of the trial [32]. von Rotz et al. (2022) gave MDD patients a single 0.215 mg/kg dose of psilocybin with therapy. Although they reported a mean within-group change of −0.35 in patient suicidal ideation as measured via the clinician-administered C-SSRS-ISS (95% CI = −0.10 to 0.71; d = 0.43; *p* = 0.13), they also report nonsignificant differences between experimental and placebo groups (F_(1,50)_ = 1.40; *p* = 0.24) [31].

##### TRD

Goodwin et al. (2022) conducted an RCT to observe the effects of a single, randomized dose of psilocybin at 1 mg (*n* = 79), 10 mg (*n* = 75), or 25 mg (*n* = 79) with psychological support in TRD patients. They used the clinician-administered C-SSRS and considered suicidal ideation with intent or endorsement of the suicidal behavior section of the scale as a serious adverse event (AE) [19]. Throughout all 12 weeks, participants exhibiting suicidal ideation or behavior were higher in the 25 mg and 10 mg groups, relative to the 1 mg group [19]. Twenty-one (27%) patients in the 25 mg group, twenty-seven (36%) in the 10 mg group, and nineteen (24%) in the 1 mg group showed suicidal ideation (passive or active with no intent to plan) at baseline [19]. From baseline to week 3, 11 (13.9%) in the 25 mg group, 13 (17.3%) in the 10 mg group, and 7 (8.9%) in the 1 mg group exhibited increased suicidal risk [19]. Of these patients, some reported serious, suicide-related AEs: two (4.5%) in the 25 mg group (suicidal ideation with intent), two in the 10 mg (7%) group (suicidal ideation with intent), and no patients in the 1 mg group [19]. From week 3 to week 12, 12 (15.2%) in the 25 mg, 12 (16.0%) in the 10 mg, and 12 (15.2%) in the 1 mg dose groups exhibited worsened suicidality. Here, serious suicide-related AEs were reported by three patients in the 25 mg group (three endorsement of suicidal behavior section of C-SSRS) and one in the 10 mg group (one suicidal ideation with intent). As participants at clinically significant risk for suicide were excluded from this study, the adverse events reported here are not part of longstanding behavioral trends [19].

##### Anorexia Nervosa

Peck et al. (2022) examined the safety of a single 25 mg dose with psychotherapy in 10 women with anorexia nervosa and reported no increases in suicidal risk as per the C-SSRS [30].

#### 3.3.2. Open-Label Trials with Suicide as a Safety or Secondary Outcome

##### TRD

Carhart-Harris et al. (2018) conducted their own open-label trial with TRD patients, giving them two oral doses of psilocybin at 10 mg and 25 mg. Using the Quick Inventory of Depressive Symptomatology-Suicidality item (QIDS-SI; out of three) and the Hamilton Depression Rating Scale-Suicidality item (HAM-D-SI; out of four), they report significant reductions in QIDS-SI scores for suicide risk from baseline to 1 week (−0.9, 95% CI = −0.4 to −1.4; *p* < 0.002) and 2 weeks (−0.85, 95% CI = −0.4 to −1.3; *p* = 0.004) post dosing [34]. Scores at 3 weeks (−0.8, 95% CI = −0.25 to −1.3, *p* = 0.01) and 5 weeks (−0.7, 95% CI = −0.22 to −1.2, *p* = 0.01) show trend reductions but were non-significant [34]. Ellis et al. (2024) also conducted an open-label trial using a 25 mg dose with psychotherapy among 15 military veterans with TRD, most (73%, *n* = 11) of whom had PTSD [37]. Using the clinician-administered C-SSRS as part of their safety assessment, they reported no increases in suicidal ideation from baseline and that no suicidal behavior was present throughout the follow-up period [37].

##### BD-II

Aaronson et al. (2024) conducted an open-label trial for the use of 25 mg of psilocybin with psychotherapy for major depressive episodes in patients with bipolar type-II disorder (BD-II) [36]. Study clinicians assessed suicidal risk using the C-SSRS-IIS as part of their safety measures and found that the main effect on timepoint was non-significant. They also reported that no patients attempted or completed suicide at any timepoints during the study [36].

##### OLTAS

Anderson et al. (2020) worked with older long-term acquired immunodeficiency syndrome survivors (OLTAS) with moderate to severe demoralization, as quantified by a score of ≥9 on the Demoralization Scale-II [35]. Patients were given 0.3–0.36 mg/kg doses of psilocybin with group psychotherapy. While the investigators reported no changes in C-SSRS-IIS scores over time nor any suicidal behavior during the intervention period, one patient (out of eighteen) attempted suicide at their 3-month follow-up [35].

#### 3.3.3. Secondary Analyses in Cancer Patients with Adjustment Disorder

Ross et al. (2021) conducted a secondary analysis assessing patient suicidal ideation in their 2016 trial where they administered a single 0.3 mg/kg dose of psilocybin with psychotherapy in cancer patients who were diagnosed with adjustment disorder with anxiety ± depression, acute stress disorder, generalized anxiety disorder (GAD), or anxiety disorder due to cancer [33]. Patients received psilocybin and placebo niacin (250 mg) in a crossover design. They used their own composite SI (suicidal ideation) measure, comprised of item nine of the Beck Depression Inventory-II (BDI-II) and item nine of the Brief Symptom Inventory (BSI) [33]. Ross et al. reported a significant within-group reduction of SI at 6.5 months from baseline (*p* < 0.001) but did not observe significant between-group effects [33].

#### 3.3.4. Qualitative Studies in Previous Healthy Psilocybin Users

Carbonaro et al. (2016) conducted a cross-sectional study asking previous healthy psilocybin users to rate their most distressing experience while on the drug [47]. Participants responded qualitatively to questions made by the investigators, some of which asked about suicidality. Out of 1993 respondents, six reported complete remission of suicidal thoughts after taking psilocybin (timeline not specified), whereas five reported increased suicidal thinking and behaviors during their experience [47]. Increased suicidality outcomes were reported as attempt to overdose and waking up in an intensive care unit (one participant), attempting to shoot themselves in the head (one participant), pre-existing depression exacerbated by psilocybin and leading to suicide attempt (one participant), and increased salience of suicidal thoughts during the experience (three participants) [47]. The context of psilocybin use (i.e., alone, one other person, small group, large group) was not reported in relation to suicidality outcomes.

### 3.4. MDMA-Assisted Therapy

Four RCTs and two cross-sectional studies examined the effects of MDMA-assisted therapy (MDMA-AT) on suicide risk as well as different suicide-related outcomes (i.e., ideation intensity, behavior).

#### 3.4.1. RCTs Assessing Suicidality as a Safety Measure in PTSD Patients

Mithoefer et al. (2018) conducted a double-blinded RCT in PTSD patients who received MDMA at a dose of 30 mg, 75 mg, or 125 mg with psychotherapy, followed by a supplemental half dose [38]. After the final RCT endpoint, the 30 mg and 75 mg groups were invited to participate in a 125 mg open-label crossover for three more sessions. The investigators stated in their safety assessment that by all post-treatment endpoints, the percentage of participants reporting suicidal ideation and behavior was reduced compared with baseline lifetime and pretreatment reports, as measured by the clinician-administered C-SSRS [38]. They also reported that there were no treatment-emergent reports of suicidal ideation [38]. Olatora et al. (2018) also ran their own double-blinded RCT with an open-label phase, where they administered to PTSD patients 40 mg, 100 mg, or 125 mg of MDMA with psychotherapy and with a supplemental half dose [39]. After the RCT endpoint, patients received either one (100 mg and 125 mg groups) or three (40 mg group) doses of 100–125 mg MDMA in an open-label crossover. While the investigators reported higher clinician-administered C-SSRS scores in the 100 mg and 125 mg groups, they did not exclude for past suicidal thinking during patient recruitment [39]. Therefore, unlike most similar studies, 28.6% of treatment-receiving participants were already impacted by symptoms of suicidal behavior before participating, and it is unclear to what extent MDMA or their medical history attributed to trends in C-SSRS scores [39]. Mitchell et al.’s (2021) RCT administered two doses of MDMA to PTSD patients through two sessions (first session: 80 + 40 mg half dose; second session: 120 + 60 mg half dose) with psychotherapy [40]. MDMA, when compared to a placebo, did not increase suicidal risk in patients with PTSD at all follow-up timepoints, as measured by a study clinician using the C-SSRS intensity of ideation subscale in their safety assessment [40]. Mitchell et al. ran another RCT in 2023 administering MDMA to PTSD patients using the same dosing regimen and found that from baseline to end of intervention, MDMA-AT did not increase suicidal risk relative to placebo in patients as scored by the clinician-administered C-SSRS [22].

#### 3.4.2. Cross-Sectional Studies: Survey Data in Non-Clinical Populations

Celine et al. (2019) looked at France’s national survey on health and drug use, ESCAPAD, and found that among French adolescents, ecstasy use is associated with increased suicide risk relative to non-users ([odds ratio] OR = 2.42, [adjusted OR] aOR = 2.74; [no *p*-values reported]) [48]. Here, adjusting the OR is accounting for covariates that influence the outcome variable, i.e., suicide risk, and is therefore controlling for confounding effects. Kim et al. (2011) conducted their own cross-sectional study using multinomial logistic regression analysis on data from the United States National Survey on Drug Use and Health (NSDUH), where, while not adjusted for confounders, they found that ecstasy use among American adolescents was associated with higher suicidal ideation and attempt compared to non-use (ideation OR: 1.5 [*p* ≤ 0.05], attempt OR: 5.5 [*p* < 0.001]) [49].

### 3.5. DMT and Ibogaine

In total, two prospective longitudinal (one cohort and one single-case), one retrospective longitudinal, and two secondary analyses (one of a RCT and one of an open-label trial) provided reports on the effect of DMT and ibogaine on suicidal behavior.

#### 3.5.1. Observational Studies in Persons with Military Trauma or PTSD

Davis et al. (2023) collected prospective data from a clinical treatment program about an open-label trial treating trauma-exposed military veterans. Patients were treated with 10 mg/kg ibogaine, followed by three doses of 5-Meo-DMT, at 5 mg, 10 mg, and 15 mg, accompanied by psychotherapy [41]. Fourth (30 mg) and fifth doses (45 mg) were administered if the patient did not reach an altered state of consciousness. The investigators reported a −0.53 (*p* < 0.01) decrease in the DSI-SS (Depressive Symptom Inventory-Suicide Subscale; out of 0 to 12) between baseline and one month post treatment [41]. Davis et al. (2020) also conducted a longitudinal, retrospective study using the same treatment regimen and found a −2.3 (*p* < 0.0001) decrease in DSI-SS scores from one month pretreatment to one month post treatment in military veterans with cognitive and physical trauma [42]. Ragnhildstveit et al. (2023) followed a 23-year-old woman with PTSD, administering to her a bufotoxin extract containing 10–15 mg of DMT. Immediately, within 24 h and for 12 months following treatment, the patient’s Beck Hopelessness Scale (BHS; out of 20) scores dropped below the cut-off for suicidal ideation and behavior (> 9) [43]. The investigators considered this drop a significant and large reduction in the patient’s suicide risk.

#### 3.5.2. Secondary Analyses in MDD Patients

Zeifman et al. (2019) conducted a secondary analysis of an RCT for treatment-resistant patients with unipolar MDD [44]. They assessed baseline patient characteristics using the MADRS-SI for a population that either received a placebo or a single 1 mL/kg dosing of ayahuasca (containing 0.36 mg/mL of DMT) with no psychotherapy. They report moderate between-group effects for decreases in MADRS-SI scores at one day (d = 0.58, 95% CI = −1.32 to 0.17), two days (d = 0.56, 95% CI = −1.30 to 0.18), and seven days (d = 0.67, 95% CI = −1.42 to 0.08) after intervention [44]. Within the ayahuasca group, there were large effect sizes for decreases in MADRS-SI at one day (d = 1.33; 95% CI 1.25 to 3.18; *n* = 14), two days (d = 1.42; 95% CI 1.50 to 3.74; *n* = 13), and seven days (d = 1.19; 95% CI 1.21 to 3.50; *n* = 14) [44]. Zeifman et al. (2021) conducted another secondary analysis on an open-label trial for MDD patients receiving 2.2 mL/kg dose of ayahuasca (itself containing 0.8 mg/mL of DMT) with no psychotherapy and found rapid reductions in suicidality, as measured through MADRS-SI scores, that remained 21 days after intervention [45].

### 3.6. LSD

One RCT and two cross-sectional studies examined LSD effects in patient and non-clinical populations, respectively.

#### 3.6.1. RCT for Patients with Anxiety Associated with Life-Threatening Disease

Gasser et al. (2015) ran an RCT for patients with anxiety associated with life-threatening disease, giving them two doses of 200 μg LSD with psychotherapy in 4-to-6-week intervals. At their 12-month follow up, 33% of patients reported “no more suicidal thoughts” and “less depressed feelings” [46].

#### 3.6.2. Cross-Sectional Studies: Retrospective Survey Data in Non-Clinical Populations

Han et al. (2022) conducted multivariable analyses using NSDUH data from 2015 to 2019 and found that past-year LSD use, relative to non-use, was associated with mildly elevated suicidal ideation among adolescents (OR = 2.4 [no *p*-values reported]). They controlled for confounding variables and reported an adjusted OR of 1.2 (no *p*-value reported) [50]. Yockey et al. (2019) examined the 2017 NSDUH results and also used multivariable analyses to conclude that lifetime LSD use among adolescents was significantly associated with more frequent thoughts about suicide (OR = 2.46 [*p* < 0.001], aOR = 1.381 [*p* < 0.001]) [51].

### 3.7. Not Specified/Multiple Psychedelic Studies

From our search, ten cross-sectional, two longitudinal, and one secondary analysis (of two longitudinal studies) examined the effects of psychedelics but either used multiple psychedelics or did not specify which psychedelics they assessed.

#### 3.7.1. Longitudinal Studies

Argento et al. conducted two overlapping longitudinal studies using a cohort of women belonging to Vancouver, Canada’s An Evaluation for Sex Workers Health Access (AESHA) cohort [54,55]. At baseline, they excluded any participants who reported previous suicidal thoughts or attempts in this analysis. They then followed participants annually for several years and later tested for associations between changes in suicidality and psychedelic use (e.g., LSD, MDMA, and psilocybin) [54,55]. Argento et al. (2017) used AESHA data from January 2010 to August 2014 and found that lifetime psychedelic use was independently associated with reduced suicidality in a population of sex workers after adjusting for covariates (OR = 1.00 [*p* = 0.995], aOR = 0.40 [*p* = 0.036]) [54]. In their follow-up study, Argento et al. (2018), using data from January 2010 to February 2017, looked at associations between psychedelic use and suicidal behaviors in users of different drugs [55]. They observed that psychedelic use moderated suicidal ideation in prescription opioid users ([OR = psychedelic use vs. no psychedelic use]; OR = 0.69 vs. 2.91 [*p* = 0.016]; aOR = 0.36 vs. 2.59 [*p* = 0.036]) and cocaine users (OR = 0.39 vs. 4.69 [*p* = 0.001] [55]. While not significant, they also tested for the effect of psychedelic use in crack users (OR = 1.31 vs. 4.08 [*p* = 0.326]), crystal meth users (OR = 1.98 vs. 4.51 [*p* = 0.206]), and heroin users (OR = 0.99 vs. 2.15 [*p* = 0.170]) [55].

#### 3.7.2. Cross-Sectional Studies: Retrospective Survey Data in Non-Clinical Populations

Guigovaz et al. (2024) used regression analyses on NSDUH data from 2008 to 2020 and found that hallucinogen use, (e.g., MDMA, LSD, psilocybin) among adolescents is not significantly associated with increased suicidal ideation, planning, or attempt (OR = 0.46, 0.56, and 1.14 respectively; [no *p*-values reported]) [62]. Sexton et al. also conducted analyses using the NSDUH [56,57]. In 2019, Sexton et al. looked at the NSDUH from 2008 to 2016 and found that lifetime novel psychedelic use (e.g., 2-[4-Bromo-2,5-dimethoxyphenyl]-ethanamine; 2C-B, 2,5-dimethoxy-4-iodophenethylamine; 2CI) compared to lifetime classic psychedelic use (e.g., LSD, mescaline, DMT, psilocybin) was associated with increased odds of suicide ideation (OR = 1.4 [*p* = 0.0180]), planning (OR = 1.6 [*p* = 0.0285]), and attempt (OR = 1.2 [*p* = 0.6310]) [56]. They also found that lifetime novel psychedelic use, compared to no lifetime psychedelic use, was associated with increased odds of suicide ideation (OR = 1.3 [*p* = 0.0749]) and planning (OR = 1.4 [*p* = 0.1196]) but not attempt (OR = 0.9 [*p* = 0.8813]) [56]. Novel psychedelics were defined as serotonin 2A receptor (5HT2AR) agonists with similar pharmacokinetics and pharmacodynamics to classic psychedelics [56]. In 2020, Sexton et al. analyzed the NSDUH from 2008 to 2017 [57]. They focused on associations between suicide and either lifetime classic tryptamine or novel phenylethylamine use. When adjusting for covariates, while they found that increased past-year suicidal thinking was not associated with lifetime classic tryptamine use (aOR = 0.79 [no *p*-value reported]), they did observe an association with novel phenylethylamine use (aOR = 1.60 [no *p*-value reported]) [57]. Hendricks et al. (2015) also looked at the NSDUH and found that in 2008–2012, lifetime classic psychedelic use, relative to no use, was associated with reduced past-year suicidal thinking (OR = 0.86 [no *p*-value reported]), planning (OR = 0.71 [no *p*-value reported]), and attempt (OR = 0.64 [no *p*-value reported]) [53]. Here, odds ratios are not adjusted for confounders. Jones et al. (2022) looked at the NSDUH from 2008 to 2019 and reported that neither MDMA or psilocybin are associated with increased suicidal planning (OR = 0.9 [*p* = 0.01]), thinking (MDMA: OR = 0.88 [*p* = 0.05]; psilocybin: OR = 0.88 [*p* = 0.08]), or attempt (MDMA: OR = 1.00 [*p* = non-significant; ns]; psilocybin: OR = 0.85 [*p* = 0.07]) [25]. However, unlike MDMA and psilocybin, LSD was associated with increased odds of suicidal thinking (OR = 1.07 [*p* = 0.05]) [25]. Again, odds ratios were not adjusted for confounders. Another article published by Jones et al. in 2022 looked at NSDUH data from 2004 to 2019 [60]. They report that while psilocybin use is associated with reduced suicidal thoughts (aOR = 0.84 [*p* ≤ 0.05]) and behaviors (suicidal planning: aOR = 0.78 [*p* ≤ 0.05]; suicidal attempt: aOR = 0.77 [*p* ≤ 0.05]), LSD use is associated with increased suicidal thinking (aOR = 1.19 [*p* ≤ 0.05]) and behaviors (suicidal planning: aOR = 1.36 [*p* ≤ 0.05]; suicidal attempt: aOR = 1.23 [*p* ≤ 0.05]) [60]. MDMA, peyote, and mescaline were examined as well but were nonsignificant and not distinctly associated with suicidal ideation or behavior [60]. Yang et al. (2022) looked at associations of LSD, tryptamine (DMT/AMT [a-methyltryptamine]/Foxy [5-methoxy-diisopropyltryptamine]), Salvia divinorum, and MDMA use with suicidal thinking, planning, and attempt from NSDUH data (2015 to 2020) [61]. Compared to non-users, LSD was associated with greater suicidal ideation (OR = 1.21 [*p* < 0.001]), planning (OR = 1.14 [*p* = ns]), and attempt (OR = 1.27 [*p* = ns]) [61]. Where Salvia divinorum use was associated with even greater ideation (OR = 1.41 [*p* < 0.05]), planning (OR = 1.74 [*p* = ns]), and attempt (OR = 2.05 [*p* = ns]) than LSD, ecstasy was associated with lesser ideation (OR = 0.86 [*p* < 0.05]), planning (OR = 0.80 [*p* = ns]), and attempt (OR = 0.83 [*p* = ns]) [61]. Tryptamine effects on suicidal ideation (OR = 1.14 [*p* = ns]) and attempt (OR = 1.16 [*p* = ns]) were comparable to LSD, but tryptamines were associated with increased suicidal planning (OR = 1.81 [*p* < 0.01]) [61]. Desai et al. (2022) used data from the Youth Risk Behavior Surveillance System (YRBSS) by the United States Centers for Disease Control and Prevention, which targets school-going adolescents [59]. The investigators found that relative to non-users, hallucinogen-(e.g., LSD, PCP [phencyclidine], mescaline, or mushrooms)-using high schoolers more often considered suicide (OR = 1.31 [*p* = 0.03]), made a suicide plan (OR = 1.44 [*p* = 0.03]), attempted suicide (OR = 1.15 [*p* = 0.395]), or had an injurious suicide attempt (OR = 1.39 [*p* = 0.118]) [59]. Importantly, these associations were not adjusted for confounders. Wong et al. used the YRBSS from 2001 to 2009 to examine if hallucinogen and ecstasy use were associated with suicidal behaviors [52]. They found that hallucinogen-using high schoolers, compared to non-users, exhibited higher suicide ideation (OR = 3.3, aOR = 1.8; [both *p* ≤ 0.0001]), planning (OR = 4.0, aOR = 1.0; [both *p* ≤ 0.0001]), suicide attempt (OR = 4.9, aOR = 2.1; [both *p* ≤ 0.0001]), or severe suicide attempt (OR = 10.4, aOR = 3.4; [both *p* ≤ 0.0001]) [52]. Interestingly, ecstasy use was also positively associated with such parameters, but to a reduced extent (ideation: OR = 3.1, aOR = 1.6 [both *p* > 0.0001]; planning: OR = 4.3, aOR = 1.5 [both *p* > 0.0001]; attempt: OR = 5.0, aOR = 1.9 [both *p* > 0.0001]; severe attempt: OR = 10.7, aOR = 2.9 [both *p* > 0.0001]) [52]. Soboka et al. (2024) examined Nova Scotia’s Mental Health and Addiction (MHA) intake program data from 2020 to 2021 and tested participant data for associations with suicide risk [63]. They considered past suicide attempts, suicidal thoughts two weeks before the interview, and suicidal ideation during the interview in defining suicide risk. They report that psychedelic use, when adjusted for covariates, was positively associated with mild (aOR = 2.04 [*p* < 0.001]) and even moderate/high (aOR = 3.54 [*p* < 0.001]) suicide risk [63].

#### 3.7.3. Secondary Analyses

Zeifman et al. (2020) performed a secondary analysis on two longitudinal, observational studies [58]. They used a composite suicidal ideation score (composite SI) made of the single-item QIDS-SI and the five-item Suicidal Ideation Attributes Scale (SIDAS; out of 50), ranging from 1 to −1. In their first study, Zeifman et al. used a convenience sample of participants planning to use a psychedelic of choice (e.g., psilocybin, LSD, ayahuasca, 5-Meo-DMT, Salvia divinorum, mescaline, or ibogaine) and reported that relative to baseline, psychedelic use was associated with significant reductions in suicidal ideation at 2 weeks (0.48 to −0.19 [*p* < 0.001]) and 4 weeks (0.48 to −0.47 [*p* < 0.001]) [58]. In the second study, participants planned to use psychedelics in the presence of a facilitator: an individual who would guide them through the process. Once again, psychedelic use was associated with a significant reduction in suicidal ideation from baseline to 4 weeks post psychedelic use (0.16 to −0.28 [*p* < 0.001]) [58].

## 4. Discussion

In this systematic review, we evaluated the effects of psychedelics on suicidal-related outcomes using 39 studies. Psilocybin was studied in multiple trials focusing on its effects on suicidal ideation and behavior, primarily in patients with depression and other psychiatric conditions. In summary, while clinical trials have supported the potential of psychedelics, especially psilocybin, in reducing suicidality in patients with psychiatric conditions, findings from observational and cross-sectional studies on general or adolescent populations suggested mixed or negative associations. However, these studies are limited by unknown confounding variables, such as the potential for increased substance use among individuals with mental health conditions or the reverse association, where those with suicidality may engage in greater substance use. Additionally, the lack of baseline data or detailed population characteristics severely limits the interpretability of these findings regarding the effects of psychedelics on suicidality, underscoring the need for further rigorous investigation.

Suicidality is a multifaceted and complex phenomenon that can manifest across a range of psychiatric disorders, including mood disorders, anxiety, PTSD, substance use disorders, and chronic pain [64]. While most pharmacological treatments are designed to target the primary disorder with the assumption that improving the underlying condition will also alleviate suicidality, this approach may be overly reductive. Suicidality may persist even in the absence of other symptoms, suggesting that treating the primary disorder may not always be sufficient to address the risk of suicide [65]. Consequently, it is critical to reconsider the assumption that treating the underlying condition inherently resolves suicidality. A more nuanced approach that evaluates suicidality as a distinct clinical target, independent of the underlying disorder, may offer more effective and tailored therapeutic interventions. This is analogous to the use of hypnotic medications to address sleep disturbances, irrespective of the underlying psychiatric condition, and could lead to more focused strategies for managing suicidality in clinical practice.

Several RCTs have reported positive effects of psychedelic administration on suicide risk, with most trials finding moderate between-group effect sizes for reductions in suicidality. Some studies reported rapid effects of psychedelics on suicidal ideations, potentially similar to ketamine [66], which is an important advantage given the limitations of current treatments due to their delayed onset of action. However, the limited reporting on the timing of suicide outcome assessments prevents definitive conclusions. Antidepressants and ECT typically take up to two weeks to show therapeutic effects [67,68], making psychedelics a critical alternative for patients with suicidal ideations without intent or plan, offering a potentially life-saving intervention when time is of the essence. The mechanisms by which psychedelics may reduce suicidality are complex and multifaceted, with one primary mechanism being the promotion of neuroplasticity, enhancing the brain’s ability to process and reframe negative beliefs and emotional experiences associated with suicidality [69,70]; however, these effects are closely influenced by factors such as set (mindset), setting (environment), and therapeutic integration [69,70]. Importantly, combining psychedelics with evidence-based therapies such as CBT may offer additional benefits [71]. Meta-analytic evidence suggests that CBT reduces suicide attempts by half in those who attempted suicide in the previous six months, making it a valuable adjunctive or follow-up intervention [72]. Moreover, psychedelics could be leveraged to address potentially modifiable factors associated with higher suicide risk, such as hopelessness, anxiety, impulsivity, psychotic symptoms, and the impact of stressful life events (e.g., financial stress, victimization) [73]. By promoting a shift in perspective on life stresses and alleviating emotional burdens, psychedelics, when combined with therapies like CBT, may more effectively target these underlying factors, further supporting reductions in suicidality [74]. Future studies should investigate the efficacy of integrated psychedelic-assisted therapy approaches to maximize therapeutic outcomes in this high-risk population.

Psychedelics also increase connectivity in brain regions involved in emotional regulation and self-reflection, facilitating emotional processing and alleviating feelings of hopelessness and despair [75]. Additionally, reductions in experiential avoidance—identified as an indirect effect in a clinical trial and as a correlate in two naturalistic studies—may contribute to decreases in suicidality by enabling individuals to confront and process difficult thoughts and emotions rather than suppressing them [58,76]. Psychedelics enable profound emotional experiences that allow individuals to confront unresolved trauma and suppressed emotions, potentially leading to emotional catharsis [77]. This is thought to be mediated by serotonin system interactions, particularly through the 5-HT2A receptor, which plays a crucial role in mood regulation [78]. The therapeutic context, which typically involves supportive, guided environments, also contributes to the reduction in suicidality by fostering trust, safety, and emotional processing, potentially leading to shifts in perspective and enhanced meaning or connectedness. The combination of psychedelics’ unique mechanisms of action, along with their rapid therapeutic effects, presents a promising option for individuals at high risk of suicide [79]. However, it is important to note that individuals at high suicide risk, including those with a history of attempts reported during screening, have been systematically excluded from included studies, limiting the generalizability of current findings to this population. Additionally, BPD has also been underrepresented in these trials, despite the elevated suicide risk associated with this group [79]. Further, while psychedelics offer rapid reductions in symptoms, the inconsistent reporting of safety outcomes across trials represents a significant limitation, particularly in studies addressing suicide outcomes. Specifically, many trials rely on scales designed to measure broader psychiatric disorders rather than directly assessing suicidality. When suicidality is evaluated, it is often through single-item measures or scales with only 2–3 questions, which fail to capture the complexity of suicidality, including its chronicity, acuity, risk level, and predisposing factors [72]. Additionally, the timing of these measurements is not always consistent, leaving uncertainty about when suicidal outcomes were assessed in relation to the psychedelic intervention. Another concern is the ambiguous categorization of suicidal outcomes—whether they are treated as adverse effects or primary outcomes [72]. If considered adverse effects, suicidality may not be systematically monitored or directly inquired about, risking underreporting when left to participant-initiated disclosures. This lack of standardization in assessing and reporting suicidal outcomes undermines a comprehensive evaluation of the risk-benefit profile of psychedelics. For comparison, ketamine and esketamine demonstrate significant short-term benefits, but their effects are often transient, requiring repeated dosing, and come with their own safety considerations [13]. Therefore, further research is essential to identify which individuals are most likely to benefit from psychedelics, to evaluate their safety in high-risk populations, and to address the caveats associated with their clinical application. Our search of clinicaltrials.gov yielded only one trial with a primary outcome focused on suicide prevention, underscoring the need for additional studies in this area.

Suicidal ideation and attempts are assessed in clinical trials as a safety measure due to concerns about the potential for increased suicidal thoughts following psychedelic administration. The C-SSRS is the most commonly used tool to evaluate suicidal ideation in these studies. While the majority of trials report no increase in suicidal risk during the study period, some severe adverse events, including suicidal ideation following psychedelic use, have been documented [79]. Additionally, one study has reported death by suicide following psilocybin administration [80]. Prior to their death by suicide, the participant exhibited no signs of behavioral impairment and showed no adverse effects during follow-up later that day or in the subsequent days [80]. However, the authors noted that this was not attributed to the intervention. In clinical trials, the controlled environment and support from trained professionals are intended to mitigate these risks, yet the unpredictable nature of psychedelic experiences means that some individuals may react in ways that are difficult to anticipate. As such, ongoing monitoring and individualized care are crucial components of ensuring participant safety, particularly for those with a history of mental health issues. Increased suicidal ideations, planning, and attempts have also been reported in population-based studies, though these findings have been inconsistent. Some studies suggest that psychedelics were associated with increased odds of suicidal behaviors in certain individuals, while others reported no significant association, and some demonstrated reduced suicidal behaviors following psychedelic use. These mixed results may be due to several factors, such as differences in study design, participant characteristics, and the specific contexts in which psychedelics are used. The intensity of the psychedelic experience itself—often involving profound emotional and psychological shifts—can vary widely among individuals, which may influence the risk of adverse outcomes. These mixed findings highlight the complexity of the relationship between psychedelics and suicidality, suggesting that further research is needed to clarify the potential risks and identify factors that may increase vulnerability to adverse outcomes. Factors such as prior mental health conditions, personal history of trauma, and the setting in which psychedelics are administered may all play a role in determining the effects on suicidality. As a result, personalized treatment approaches and careful risk assessment are essential for ensuring participant safety, particularly for those with pre-existing mental health concerns.

This systematic review added to the literature by synthesizing existing research on the effects of psychedelic therapies—specifically psilocybin and other serotonergic psychedelics—on suicidality in individuals with psychiatric disorders. By focusing on RCTs, observational studies, and other relevant data, this review evaluated the potential of psychedelics as a treatment for suicide risk. It highlighted the methodological gaps in current research, such as the exclusion of high-risk populations, inconsistent definitions of suicidality, and limited longitudinal data. Additionally, this review discussed the implications for clinical practice and future research directions, providing an evidence-based foundation for the safe integration of psychedelic therapies into psychiatric care, particularly for individuals at high risk for suicide. Given these findings, it is clear that psychedelic therapies hold promise for addressing suicidality in psychiatric patients, particularly when combined with psychotherapy. However, due to methodological limitations and a lack of data from high-risk populations, it is premature to implement these therapies as standard practice. Further research is required to better understand their long-term efficacy, safety, and optimal treatment protocols. Only through rigorous clinical trials that include high-risk individuals and consistent outcome measures can we determine the potential role of psychedelics in clinical settings.

### Limitations

The limitations of this review include several factors that may affect the validity and generalizability of the results. First, the sample size is insufficient to detect significant effects, limiting the ability to generalize findings to broader populations. Selection bias is another concern, as participants may not be randomly selected or fully representative of the target population. Additionally, higher-risk patients are often excluded, which limits the study’s external validity and generalizability. Confounding variables, such as unmeasured factors influencing the outcomes, could also distort the findings. Moreover, the short duration of the study limits the ability to assess long-term effects or trends, which may be important in understanding sustained outcomes. If a study is cross-sectional, it can only establish associations, not causality, which is a significant limitation. Furthermore, the absence of subgroup analyses for specific psychedelics and populations limited the ability to assess the nuanced effects across different types of psychedelics and varied populations. The lack of data suitable for a meta-analysis prevented a more comprehensive synthesis of results across studies. Additionally, publication bias may lead to an overrepresentation of positive findings, as studies with negative or null results are less likely to be published, which may influence the conclusions drawn. These limitations should be carefully considered when interpreting the findings. Ethical considerations, accessibility, and scalability of psychedelic therapies are critical issues that must be addressed in future research. The potential for these therapies to be widely implemented in clinical settings depends on overcoming significant barriers related to their cost, regulatory approval, and the need for trained professionals to administer them safely. Additionally, the mechanisms by which psychedelics exert their therapeutic effects remain not fully understood, highlighting a need for further research into their pharmacodynamics and neurobiological mechanisms. Furthermore, although certain adverse effects have been identified, there is a clear gap in the systematic evaluation of potential long-term risks associated with psychedelic use, such as exacerbation of mental health symptoms or emergence of new psychological issues. These concerns must be addressed to ensure the safe and effective use of psychedelics in treating psychiatric disorders.

## 5. Future Directions

Future research should focus on well-designed longitudinal studies that consistently define and assess suicide-related outcomes, account for confounding factors, and evaluate long-term safety and efficacy. Expanding inclusion criteria to encompass high-risk populations will be critical in determining whether psychedelics can be safely and effectively used for suicide risk reduction. Standardizing adverse event reporting, including suicidality, is essential to clarify potential risks. Addressing these gaps will provide a clearer understanding of whether psychedelics have a meaningful role in managing suicidality in clinical and real-world settings.

## 6. Conclusions

While clinical trials suggest that psychedelics, particularly psilocybin, may reduce suicide-related outcomes in individuals with psychiatric disorders, the evidence remains inconclusive. Reductions in suicidality appear closely linked to improvements in comorbid symptoms such as depression, PTSD, and anxiety, rather than a direct effect on suicidality itself. Methodological limitations, including inconsistent definitions of suicide-related outcomes, reliance on single-item measures, and variability in outcome assessment timing, weaken the strength of current findings. Additionally, observational and cross-sectional studies indicate more variable results, with some evidence suggesting increased suicidality in certain populations. The exclusion of high-risk individuals from clinical trials further limits the generalizability of these findings. At present, there is insufficient evidence to support the clinical use of psychedelics for suicide prevention, and further research is needed to determine their safety and efficacy in this context. Given these factors, it remains premature to conclude that psychedelics have a definitive role in mitigating suicidality.

## Figures and Tables

**Table 1:** Characteristics of included intervention-based studies.

Author	Country	Study Design	Participants	Population	Relevant Exclusion Criteria	Intervention	Psychotherapy Principle	Outcome Measure	Results	Conclusion
Psilocybin										
Carhartt-Harris, 2021 [28]	United Kingdom	RCT	Psilocybin group: *n* = 30, 11% female, mean age of 43.3 Placebo group: *n* = 29, 9% female, mean age of 39.1	MDD	Currently or previously diagnosed with psychotic disorder, family member with psychotic disorder, history of serious suicide attempts requiring hospitalization, or BPD	Psilocybin group: 2 doses of 25 mg Psilocybin3 weeks apart + 6 weeks of daily oral placebo Placebo group: 2 doses 1 mg of Psilocybin 3 weeks apart + 6 weeks of daily oral escitalopram. Both groups were given psychotherapy.	1 3-h preparation session, 2 4–6 h dosing sessions, 6 90 min integration sessions 3 integration sessions followed each dose. The first was in-person, and the second was over an online call. Length of these sessions was not specified.	SIDAS	Assessed as a secondary outcome. Change from baseline to 6 weeks. *n* = 30, Psilocybin group: −2.0 (95% CI; −4.3 to 0.0) *n* = 29, Placebo group: −0.8 (95% CI; −3.4 to 2.0) Between-group difference: −1.3 (95% CI = −6.5 to −0.3) Confidence intervals were not corrected for multiple comparisons.	Suggests that psilocybin has reduced suicide risk vs. escitalopram, but the intervals for the between group comparison were not corrected.
Davis, 2021 [29]	United States	RCT	*n* = 27, 67% women, mean age of 39.8 years	MDD	Personal or family history (first or second degree) of psychotic or bipolar disorders, medically significant suicide attempt, BP-I or II	Psilocybin at 20 mg/70 kg, followed by 30 mg/kg. Accompanied with psychotherapy. Session 1: 20 mg/70 kg Session 2: 30 mg/70 kg	8 h of preparation sessions, 2 day-long dosing sessions, no integration Immediate group received the intervention right after baseline screening, whereas the delayed group waited 8 weeks in between baseline and intervention.	Clinician-administered C-SSRS, ideation intensity subscales	Assessed as a secondary outcome. 24 completed the study. Remaining 3 opted out of study with no AE. Immediate group Baseline: 1.2 5 weeks post: 0.2 (*p* = ns) 8 weeks post: 0.2 (*p* = ns) Delayed group Baseline: 1.3 5 weeks post: 0.6 (*p* = ns) 8 weeks post: 0.5 (*p* = ns)	Psilocybin did not increase suicidal risk in MDD patients, regardless of delayed treatment.
Goodwin, 2022 [19]	United States, Czech Republic, Denmark, Germany, Ireland, Netherlands, Portugal, Canada, Spain, United Kingdom	RCT	*n* = 233, 52% female, mean age of 39.8	TRD	Current or past history of schizophrenia, psychotic disorder (unless substance induced or due to a medical condition), bipolar disorder, delusional disorder, paranoid personality disorder, schizoaffective disorder, BPD, or any serious psychiatric comorbidity, clinically significant risk for suicide	Single dose of psilocybin at either 25 mg (*n* = 79), 10 mg (*n* = 75), or 1 mg (*n* = 79) All groups were given psychotherapy.	3 preparation sessions, 1 6–8 h dosing session, 2 integration sessions (time not specified)	Clinician-administered C-SSRS	Assessed as a safety measure. While individual scores were not, the number of participants exhibiting suicidal ideation and behavior per group was provided. Throughout all 12 weeks, Participants in the 25 mg and 10 mg groups exhibited higher suicidal ideation than in the 1 mg group. Baseline: 21 in the 25 mg group, 27 in the 10 mg group, 19 in the 1 mg group showed suicidal ideation (passive or no intent to plan) Baseline to week 3: 11 in the 25 mg group, 13 in the 10 mg group, and 7 in the 1 mg group showed increased suicidal risk. Here, 2 in the 25 mg and 2 in the 10 mg groups were noted for suicidal ideation with intent. Week 3 to week 12: 12 in the 25 mg, 12 in the 10 mg, and 12 in the 1 mg groups exhibited worsened suicidality. Here, 3 in the 25 mg group endorsed items on the behavior section of the C-SRSS, and 1 in the 10 mg group exhibited suicidal ideation with intent.	No clear evidence for reductions or increases in suicide risk due to psilocybin.
Peck, 2022 [30]	United States	RCT	*n* = 10, 100% female, mean age of 28.3	Anorexia nervosa	Current or previously diagnosed psychotic disorder, substantial suicide risk, history of mania and borderline personality disorder	Single 25 mg dose of psilocybin with psychotherapy.	2 preparation sessions, 1 8 h dosing session, 2 90 min integration sessions	Clinician administered C-SSRS	Assessed as a safety measure. No increases in suicidal ideation throughout trial, and no suicidal behaviors present in follow-up visits	No evidence to suggest psilocybin increases suicidal risk.
Von Rotz, 2022 [31]	Switzerland	RCT	*n* = 52 Psilocybin *n* = 26, 61.5% female, mean age of 37.6 Placebo *n* = 26, 65.4% female, mean age of 35.9	MDD	Axis-II disorder symptoms associated with increased adverse emotional or behavioral reactions to intervention, psychosis spectrum disorders and/or mania symptoms in participants or first-degree relatives.	Single 0.215 mg/kg dose of psilocybin. Both groups were given psychotherapy.	2 1 h preparation sessions, 1 6 h dosing session, 3 1 h integration sessions	Clinician administered C-SSRS, intensity of ideation subscale	Assessed as a secondary outcome. Mean. Psilocybin Baseline: 0.50 14 days: 0.15 Change: −0.35 (*p* = 0.13, 95% CI: −0.10 to 0.71, d = 0.43) Placebo Baseline: 0.54 14 days: 0.46 Between-group difference not significant. (F[1, 50] = 1.40; *p* = 0.24)	No detectable impact of psilocybin on suicide risk.
Raison, 2023 [32]	United States	RCT	*n* = 104, 50% female, mean age of 41.1 *n* = 51 given psilocybin, 47% female, mean age of 40.4	MDD	History of psychosis or mania, active suicidal ideation with intent.	Single 25 mg dose of psilocybin All groups were given psychotherapy.	6–8 h of preparatory sessions, 1 7–10 h dosing session, 4 h of integration sessions	Clinician administered C-SSRSor MADRS-SI	Assessed as a safety measure. No suicidal or self-injurious behavior occurred during the trial. All instances of ideation were passive. One participant receiving psilocybin and five receiving niacin had an increase in C-SSRS score from baseline to end of trial.	Little evidence of Psilocybin increasing suicide risk.
Ross et al., 2021 [33]	United States	RCT, secondary analysis	*n* = 11, 63.6% female, mean age of 60.3 *n* = 6 assigned to psilocybin first, 50% female, mean age of 57.5 *n* = 5 assigned to placebo first, 80% female, mean age of 63.6	Cancer patients with either adjustment with anxiety ± depression, acute stress disorder, generalized anxiety disorder (GAD), or anxiety disorder due to cancer.	Personal or immediate family history of schizophrenia, bipolar disorder, delusional disorder, paranoid disorder, and schizoaffective disorder.	Psilocybin at 0.3 mg/kg, crossed over with placebo. One group received psilocybin at the first dosing session and placebo at the second, whereas the other group received placebo first and psilocybin second. Both groups were given psychotherapy.	3 preparation sessions, 1st dosing session, cross over session (post dose integration + preparation for dose 2) over 7 weeks, 2nd dosing session, 3 integration sessions	Composite SI score using BDI-II and BSI	Composite score out of 100. Based on summed Z-scores of item 9 of BDI-II and item 9 of BSI, SI after dose 1 Psilocybin first Baseline: 70 8 h: 40 2 weeks: 40 7 weeks: 38 Placebo first Baseline: 66 8 h: 48 2 weeks: 52 7 weeks: 48 SI after dose 2 Both groups Baseline: 62 6.5 months post: 35	Authors note “acute and sustained reductions in SI… in patients with life threatening cancer”. They report significant within-group improvements in SI.
Carhart-Harris et al., 2018 [34]	United Kingdom	Open Label	*n* = 20, 30% female, mean age of 44.1	TRD	Current or previously diagnosed psychotic disorder or immediate family member with a diagnosed psychotic disorder	Two oral doses of psilocybin at 10 mg and 25 mg with psychotherapy.	1 preparation session, 2 dosing sessions	QIDS-SI, HAM-D-SI	Relevant items taken from primary outcome measures. Reported as mean change. QIDS-SI Change at 1 week post: −0.9 (95% CI = −0.4 to −1.4,; *p* < 0.002) Change at 2 weeks post: −0.85 (95% CI = −0.4 to −1.3; *p* = 0.004) Change at 3 weeks post: −0.8 (95% CI = −0.25 to −0.13; *p* = 0.01) Change at 5 weeks post: −0.7, 95% CI = −0.22 to −1.2; *p* = 0.01) HAM-D-SI Change at 1 week post: −0.95 (95% CI = −0.58 to −1.3; *p* < 0.001)	Authors note significant reductions at week 1 and 2 post treatment and nonsignificant changes 3 and 5 weeks out.
Anderson et al., 2020 [35]	United States	Open Label	*n* = 18, 0% female, mean age of 59.2	OLTAS, Demoralization scale-II ≥ 8	Primary psychotic disorder, bipolar disorder I/II), severe major depressive episode, suicidal ideation with intent in the last three months or suicide attempt in last two years	A single 0.3–0.36 mg/kg dose of psilocybin with psychotherapy.	1 90 min preparation session, 1 8 h dosing session, 1 2 h individual psychotherapy session, 8–10 group psychotherapy sessions spread throughout 7-week trial period	Clinician-administered C-SSRS, ideation intensity subscale	Assessed as a safety measure. Mean. Baseline, week −3: 0.5 Week −1: 0.11 Day after drug, week 0: 0 Week 1 to 2: 0.11 End of treatment: 0.28 Reported no change in suicidal ideation over time, and no suicidal behavior detected during intervention.	No significant evidence to suggest increased suicide risk
Aaronson et al., 2024 [36]	United States	Open Label	*n* = 15, 60% female, mean age of 37.8	BP-II	History of BP-I disorder, schizophrenia, psychosis, delusions, paranoid, schizoaffective, or borderline personality disorder	A single 25 mg dose of psilocybin with psychotherapy.	3 preparation sessions, 1 8 h dosing session, 3 1 h integration sessions	Clinician-administered C-SSRS, ideation intensity subscale	Assessed as a safety measure. No patients attempted or committed suicide at any timepoints during the study.	No detectable impact on psilocybin-related suicide risk
Ellis et al., 2024 [37]	United States	Open Label	*n* = 15, 13% female, mean of 43.2	Veterans with TRD	Current or past psychotic disorder, bipolar disorder, or personality disorder, current significant risk for suicide or engaged in suicidal behavior in the last three months	Single dose of 25 mg psilocybin with psychotherapy.	2 60 min and 1 90 min preparation sessions, 1 6–8 h dosing session, 3 90 min integration sessions	Clinician administered C-SSRS	Assessed as a safety measure. No observed increases in suicidal ideation from baseline, and no suicidal behavior present throughout the follow-up period.	No detectable impact on psilocybin-related suicide risk
MDMA-AT										
Mithoefer et al., 2018 [38]	United States	RCT	*n* = 26, 27% female, mean age of 37.2	PTSD	BP-I	Participants received MDMA-AT with 30 mg (*n* = 7), 75 mg (*n* = 7), or 125 mg (*n* = 12) All groups were given psychotherapy.	3 preparation sessions, 2 8 h dosing sessions, 3 integration sessions. Open-label crossover, 30 mg and 75 mg groups received 3 sessions of 100–125 mg	Clinician administered C-SSRS	Assessed as a safety measure. Numbers indicate participants with any positive ideation; C-SSRS ideation scale ≥ 1. Parenthesis indicate participants with serious ideation; C-SSRS ideation scale ≥ 4. 30 mg Baseline: 5(1) 1st session Pre-Drug: 1 Post-Drug: 1 Int. 1: 0 Int. 2: 1 Int. 3: 1 2nd session Pre-Drug: 0 During-Drug: 0 Int. 1: 0 Int. 2: 1 Int. 3: 2 3rd Session Pre-Drug: 1 During-Drug: 0 Int. 1: 0 Int. 2: 0 Int. 3: 0 75 mg Baseline: 6(2) 1st session Pre-Drug: 0 During-Drug: 0 Int. 1: 0 Int. 2: 0 Int. 3: 0 2nd session Pre-Drug: 0 During-Drug: 0 Int. 1: 0 Int. 2: 0 Int. 3: 0 3rd session Pre-Drug: 0 During-Drug: 0 Int. 1: 0 Int. 2: 0 Int. 3: 0 125 mg Baseline: 11(5) 1st session Pre-Drug: 0 During-Drug: 0 Int. 1: 1 Int. 2: 2 Int. 3: 3 2nd session Pre-Drug: 1 During-Drug: 0 Int. 1: 0 Int. 2: 1 Int. 3: 2 Session 3 Pre-Drug: 0 During-Drug: 0 Int. 1: 1 Int. 2: 2 Int. 3: 2 Open label crossover. 30 mg and 75 mg groups received 100–125 mg MDMA for 3 more sessions 30 mg 4th session Pre-Drug: 0 During-Drug: 0 Int. 1: 1 Int. 2: 0 Int. 3: 0 5th session Pre-Drug: 0 During-Drug: 0 Int. 1: 0 Int. 2: 0 Int. 3: 0 6th session Pre-Drug: 0 During-Drug: 0 Int. 1: 0 Int. 2: 0 Int. 3: 0 75 mg 4th session Pre-Drug: 0 During-Drug: 0 Int. 1: 0 Int, 2: 0 Int. 3: 0 5th session Pre-Drug: 1 During-Drug: 0 Int. 1: 0 Int. 2: 0 Int. 3: 0 6th session Pre-Drug: 0 During-Drug: 0 Int. 1: 0 Int. 2: 0 Int. 3: 0	Little evidence to suggest that MDMA may reduce suicidal risk in PTSD patients. There were no treatment emergent reports of suicidal ideation.
Olatora et al., 2018 [39]	United States	RCT	Total *n* = 28, 67.9% female, mean age of 42 40 mg *n* = 6, 83.3% female, mean age of 40 100 mg *n* = 9, 66.7%, female mean age of 39.6 125 mg *n* = 13, 61.5% female, mean age of 44.6	PTSD	All personality disorders	2 separate doses of 40 mg, 100 mg, or 125 mg, followed by a supplemental half dose. All groups were given psychotherapy.	3 90 min preparatory sessions, 2 8 h dosing sessions, 3 integration sessions Open-label crossover, 100 mg and 125 mg groups received 1 session of 100–125 mg, accompanied by 3 integration sessions. 40 mg group received 3 sessions of 100–125 mg, accompanied by 1 preparatory session and 6 integration sessions.	Clinician-administered C-SSRS	Assessed as a safety measure. Numbers indicate participants with any positive ideation; C-SSRS ideation scale ≥ 1. Parenthesis indicate participants with serious ideation; C-SSRS ideation scale ≥ 4. RCT Phase 40 mg 1st session Baseline: 0 Pre-drug: 0 Post-drug: 0 Int. 1: 0 Int. 2: 1 Int. 3: 2 2nd session Baseline: 0 Pre-drug: 0 Post-drug: 0 Int. 1: 0 Int. 2: 0 Int. 3: 0 100 mg Baseline: 6 1st session Pre-drug: 5 Post-drug: 2 Int. 1: 3 Int. 2: 3 Int. 3: 5 2nd session Pre-drug: 3 Post-drug: 3 Int. 1: 3 Int. 2: 3 Int. 3: 7 125 mg Baseline: 7 1st session Pre-drug: 3 Post-drug: 0 Int. 1: 0 Int. 2: 4 Int. 3: 5 2nd session Pre-drug: 3 Post-drug: 5 Int. 1: 0 Int. 2: 7 Int. 3: 9 Open-label crossover 100 mg 3rd session Pre-drug: 3 Post-drug: 2 Int. 1: 1 Int. 2: 3 Int. 3: 3 125 mg 3rd session Pre-drug: 1 Post-drug: 3 Int. 1: 1 Int. 2: 1 Int. 3: 4 40 mg 4th session Pre-drug: 0 Post-drug: 1(1) Int. 1: 1(1) Int. 2: 0 Int. 3: 1 5th session Pre-drug: 0 Post-drug: 0 Int. 1: 0 Int. 2: 0 Int. 3: 0 6th session Pre-drug: 1 Post-drug: 0 Int. 1: 0 Int. 2: 1 Int. 3: 0	Authors observe greater suicide risk in active vs. comparator dose groups, but they did not exclude for past suicidal thinking in their patients.
Mitchell et al., 2021 [40]	United States, Canada, Israel	RCT	Total *n* = 90, 65.5% female, mean age of 40.9 MDMA-AT group *n* = 46, 58.7% female, mean age of 43.5 Placebo group *n* = 44, 72.7% female, mean age of 38.2	PTSD	Primary psychotic disorder, BP-I, dissociative identity disorder, MDD with psychotic features, personality disorders	Session 1: Dose 1: 80 mg Dose 2: 40 mg (1.5–2.5 h after after dose 1) Session 2: Dose 1: 120 mg Dose 2: 60 mg (1.5–2.5 h after after dose 1) All groups were given psychotherapy.	3 preparatory sessions, 3 8 h dosing sessions, 9 integration sessions. Dosing sessions were spaced out 4 weeks apart, and 3 integration sessions occurred after each dosing session. The first integration session was the day after dosing, and the remaining two occurred in the following 3–4 weeks.	Clinician-administered C-SSRS	Assessed as a safety measure. Numbers indicate participants with any positive ideation; C-SSRS ideation scale ≥ 1. Parenthesis indicate participants with serious ideation; C-SSRS ideation scale ≥ 4. Placebo group Baseline: 14(1) 1st Session Pre-drug: 5(1) Post-drug: 6(1) Int. 1: 5(1) Int. 2: 11(1) Int. 3: 11(1) 2nd Session Pre-drug: 7(1) Post-drug: 3 Int. 1: 4(2) Int. 2: 8(2) Int. 3: 13(2) 3rd Session Pre-drug: 2 Post-drug: 1 Int. 1: 4(1) Int. 2: 8(2) Int. 3: 9(2) MDMA-AT Baseline: 17 1st Session Post-drug: 9 Pre-drug: 2 Int. 1: 0 Int. 2: 9 Int. 3: 13 2nd Session Pre-drug: 7 Post-drug: 1 Int. 1: 3 Int.2: 5 Int.3: 11 3rd Session Pre-drug: 3 Post-drug: 0 Int. 1: 1 Int. 2: 6(1) Int. 3: 8	Compared to placebo, MDMA did not increase suicide risk in patients with PTSD.
Mitchell et al. 2023 [22]	United States, Israel	RCT	*n* = 104, 71.2% female, mean age of 39.02 MDMA-AT group *n* = 53, 60.4% female, mean age of 38.2 Placebo *n* = 51, 82.4% female, mean age of 40	PTSD	Clinically significant suicide risk and comorbid personality disorders	Session 1: 80 mg dose + 40 mg half dose 2 h later Sessions 2 and 3: 120 mg dose + 60 mg half dose 2 h later All groups were given psychotherapy.	3 90 min preparation sessions 3 8 h dosing sessions, 9 90 min integration sessions Each dosing session was followed by 3 integration sessions.	Clinician-administered C-SSRS, ideation intensity and behavior subscales	Assessed as a safety measure. Numbers indicate participants with any positive ideation; C-SSRS ideation scale ≥ 0. Parenthesis indicate participants with serious ideation; C-SSRS ideation scale ≥ 4. Placebo Baseline: 12 1st Session Pre-drug: 4 Post-drug: 6 Int. 1: 2 Int. 2: 7 Int. 3: 10 2nd Session Pre-drug: 5 Post-drug: 5 Int. 1: 3 Int. 2: 4 Int. 3: 10 3rd Session Pre-drug: 4 Post-drug: 3(1) Int. 1: 2(1) Int. 2: 3 Int. 3: 8 MDMA-AT Baseline: 13 1st Session Pre-drug: 3 Post-drug: 2 Int. 1: 2 Int. 2: 3 Int. 3: 4 2nd Session Pre-drug: 2 Post-drug: 3 Int. 1: 2 Int. 2: 4 Int. 3: 4 3rd Session Pre-drug: 4 Post-drug: 3 Int. 1: 2 Int. 2: 5 Int. 3: 11(1)	Suggests that MDMA-AT does not increase suicide risk in PTSD patients.
DMT										
Davis et al., 2023 [41]	Mexico	Longitudinal, prospective	*n* = 86, 0% female, mean age of 42.88	Trauma-exposed military veterans	Current or past psychotic spectrum disorders or BD-I, or symptoms of impaired reality testing	10 mg/kg Ibogaine + 3 doses of 5-MeO-DMT (5 mg, 10 mg, 15 mg). Fourth (30 mg) and fifth doses (45 mg) if needed. Given with psychotherapy.	Day 1: Preparation session, ibogaine dosing Day 2: Ibogaine integration Day 3: Preparation, 5MeO-DMT dosing Following days: Integration in groups and individually	DSI-SS	Baseline (*n* = 86): 1.03 1 month (*n* = 71): 0.60 3 month (*n* = 62): 0.70 6 month (*n* = 52): 0.67 Change from baseline to 1 month: −0.53 (*p* = <0.01)	Ibogaine and 5-MeO-DMT assisted psychotherapy moderately reduced suicidal ideation in trauma exposed veterans.
Davis et al., 2020 [42]	Mexico	Longitudinal study, retrospective	51 participants, 4% female, mean age of 40.4	Military veterans with cognitive and physical trauma	Current or past psychotic spectrum disorders or bipolar I disorder, or symptoms of impaired reality testing	10 mg/kg Ibogaine + 3 doses of 5-MeO-DMT (5 mg, 10 mg, 15 mg). Fourth (30 mg) and fifth doses (45 mg) if needed. Given with psychotherapy.	Day 1: Preparation session, ibogaine dosing Day 2: Ibogaine integration Day 3: Preparation, 5MeO-DMT dosing Following days: Integration in groups and individually	DSI-SS	*n* = 41 completed the study; 10 refused to respond. 1 month pre-treatment: 2.7 1 month post-treatment: 0.4 Change: −2.3 (*p* = <0.001)	Ibogaine and 5-MeO-DMT decrease suicidal ideation in traumatized military veterans.
Ragnhildstveit et al., 2023 [43]	United States	Longitudinal, prospective	*n* = 1, female, 23 years old	PTSD	NA	10−15 mg of 5-MeO-DMT via vaporized bufotoxin from *Incilius alvarius* with psychotherapy.	1 dosing session, followed by weekly follow-ups	BHS	Baseline: 17 24 h: 8 1 month: 4 3 month: 9 6 month: 3 12 month: 8	5-MeO-DMT decreases feelings of hopelessness and suicidality in the case of a 23-year-old woman with PTSD.
Zeifman et al., 2019 [44]	Brazil	Secondary analysis, RCT	Total *n* = 29, 72% female, mean age of 42.04 Drug group *n* = 14, 72% female, mean age of 39.71	Treatment resistant unipolar MDD	Imminent suicidal risk, personal or family history of schizophrenia, bipolar affective disorder, mania, or hypomania.	1 mL/kg dose of ayahuasca containing 0.36 mg/mL DMT, with no psychotherapy	1 8 h dosing session	MADRS-SI	Between-group effects 1 day: d = 0.58 (95% CI = −1.32 to 0.17) 2 days: d = 0.56 (95% CI = −1.30 to 0.18) 7 days: d = 0.67 (95% CI = −1.42 to 0.08) Ayahuasca within-group effects 1 day: d = 1.33 (95% CI = 1.25 to 3.18, *n* = 14) 2 days: d = 1.42 (95% CI = 1.50 to 3.74, *n* = 13) 7 days: d = 1.19 (95% CI = 1.21 to 3.50, *n* = 14)	Relative to placebo, ayahuasca did not significantly reduce suicidality in patients with unipolar MDD
Zeifman et al., 2021 [45]	Brazil	Secondary analysis, open label	*n* = 17, 82% female, mean age of 42.71	MDD	Presence of active psychotic symptoms, a diagnosis of bipolar or psychotic disorder, or a history of antidepressant or substance-induced mania or hypomania	2.2 mL/kg dose of ayahuasca containing 0.8 mg/mL DMT, with no psychotherapy.	1 4 h dosing session	MADRS-SI	*n* = 15; 2 participants showed 0 suicidality at baseline and were excluded from analysis Baseline: 2.40 1 day: 0.53 7 days: 0.53 14 days: 0.53 21 days: 0.33	Ayahuasca is associated with reduced suicidality among MDD patients within a day of administration, and effects last for at least 21 days post dosing.
LSD										
Gasser et al., 2015 [46]	United States	RCT	*n* = 10, 60% female, mean age of 51.1	Anxiety associated with life-threatening disease	Not mentioned	2 doses of 200 μg LSD with psychotherapy	6–8 therapy sessions in 3 months of which 2 included LSD experience	Semi-structured interview at 12-month follow-up	33% of participants reported “… no more suicidal thoughts, less depressed feelings”	No evidence to suggest that LSD alters suicidal thinking

Abbreviations: AE: adverse event. RCT: randomized controlled trial. MDD: major depressive disorder. TRD: treatment-resistant depression. PTSD: post-traumatic stress disorder. BP-I: bipolar I disorder. BP-II: bipolar II disorder. BPD: borderline personality disorder. OLTAS: older long-term acquired immunodeficiency syndrome survivor. MADRS-SI: Montgomery–Åsberg Depression Rating Scale-Suicidality Item. C-SSRS: Columbia–Suicide Severity Rating Scale. C-SSRS-IIS: C-SSRS intensity of ideation subscale. DSI-SS: Depressive Symptom Index-Suicidality Subscale. Composite SI: Composite Suicidality Item. BHS: Beck Hopelessness Scale. BDI-II: Beck Depression Inventory II. BSI: Brief Symptom Inventory. QIDS-SI: Quick Inventory of Depressive Symptomatology-Suicidality item. HAM-D-SI: Hamilton Depression Rating Scale-Suicidality item. SIDAS: Suicidal Ideation Attributes Scale. MDMA-AT: 3,4-methylenedioxymethamphetamine-assisted therapy. Int.: integration.

**Table 2:** Characteristics of included population studies.

Author	Country	Study Design	Participants	Population	Intervention	Psychotherapy Principle	Outcome Measure	Results	Conclusion
Psilocybin									
Carbonaro et al., 2016 [47]	United States	Cross-sectional	*n* = 1993, 22% female, mean age of 30	Healthy, taken psilocybin before and experienced distress during their trip	NA	NA	Qualitative responses made by researchers	5 participants reported increased suicidality, whereas 6 reported decreased suicidality	Unmonitored psilocybin use can result in distressing experiences that increase suicidality. However, a similar amount of participants report decreased suicidality after drug use.
MDMA									
Celine et al., 2019 [48]	France	Cross-sectional	*n* = 26, 351 participants, age 17	Healthy	NA	NA	ESCAPAD (no year provided)	Suicidal ideation in ecstacy/ amphetamine users vs. non users OR: 2.42 aOR: 2.74 (no *p*-value reported)	Ecstacy/amphetamine use, relative to no use, was associated with increased suicide ideation even when adjusted for covariates.
Kim et al., 2011 [49]	United States	Cross-sectional	*n* = 19,301, aged 12–17	Healthy	NA	NA	NSDUH (2000)	OR of ecstasy use and suicidal behaviors (adjusted for family and individual factors): Suicidal ideation: 1.5 (*p* ≤ 0.05) Suicide attempt: 5.5 (*p* ≤ 0.001)	Ecstacy use among adolescents was associated with increased odds of suicidal ideation and even greater odds of suicidal attempt.
LSD									
Han et al., 2022 [50]	United States	Cross-sectional	*n* = 69,916, aged 12–17	Healthy	NA	NA	NSDUH (2015–2019)	OR of past year LSD use vs. never used LSD on suicidal ideation OR: 2.4 aOR: 1.2 OR of lifetime but no past year use vs. never used LSD on suicidal ideation OR: 2.0 aOR: 1.1 (no *p*-values reported)	LSD users had a slightly higher risk of suicidal ideation than non LSD users.
Yockey et al., 2019 [51]	United States	Cross-sectional	*n* = 13,840, 51.6% female, aged 12–17	Healthy	NA	NA	NSDUH (2017)	OR of Lifetime LSD use vs. no use on thoughts about suicide OR: 2.46 (*p* < 0.001) aOR: 1.381 (*p* < 0.001)	LSD use was associated with increased thoughts of suicide
Not Specified/Multiple Psychedelic Studies									
Wong et al., 2013 [52]	United States	Cross-sectional	*n* = 73,183, 49.3% female, mean age of 16.0, school-going adolescents	Healthy	NA	NA	YRBSS (2001–2009)	OR of hallucinogen use vs. no use on: (* = *p* < 0.0001) Suicide ideation OR: 3.3 * aOR: 1.8 * Suicide plan: OR: 4.0 * aOR: 1.9 * Suicide attempt OR: 4.9 * aOR: 2.1 * Severe suicide attempt OR: 10.4 * aOR: 3.4 * OR of ecstasy use vs. no use compared to non users: (* = *p* < 0.0001) Suicide ideation OR: 3.1 * aOR: 1.6 * Suicide plan OR: 4.3 * aOR: 1.5 * Suicide attempt OR: 5.0 * aOR: 1.9 * Severe suicide attempt OR: 10.7 * aOR: 2.9 *	Hallucinogen and ecstacy use are associated with increased suicidal ideation and planning, suicide attempt, and severe suicide attempt. Notably, ecstasy has a weaker association with these parameters than hallucinogen use.
Hendricks et al., 2015 [53]	United States	Cross-sectional	*n* = 27,235 psychedelic users, 37.2% female, aged 12–17	Healthy	NA	NA	NSDUH (2008–2012)	Classic psychedelics defined as DMT, mescaline, LSD, psilocybin, peyote, OR of lifetime classic psychedelic use vs. no use on: Past year suicidal thinking: 0.86 Past year suicidal planning: 0.71 Past year suicidal attempt: 0.64 (no *p*-values reported)	Lifetime classic psychedelic use is associated with reduced suicidal thinking, planning, and attempt
Argento, 2017 [54]	Canada	Longitudinal, prospective	290 participants, 100% women	Healthy, marginalized sex workers	NA	NA	AESHA (2010–2014)	OR of lifetime psychedelic use vs. no use on suicidality: OR: 1.00 (*p* = 0.995) aOR: 0.40 (*p* = 0.036)	Psychedelic use is independently associated with reduced suicidality in a population of sex workers.
Argento, 2018 [55]	Canada	Longitudinal, prospective	340 participants, 100% women	Healthy, marginalized sex workers	NA	NA	AESHA (2010–2017)	Psychedelics defined: LSD, MDMA, psilocybin OR of psychedelic vs. no use on suicidal behavior: Prescription opioid users (psychedelic use vs. no use): 0.69 vs. 2.91 (*p* = 0.016) Cocaine users: 0.39 vs. 4.69 (*p* = 0.001) Crack users: 1.31 vs. 4.08 (*p* = 0.326) Crystal Meth users: 1.98 vs. 4.51 (*p* = 0.206) Heroin users: 0.88 vs. 2.15 (*p* = 0.170)	Psychedelic use moderated suicidality among prescription opioid, cocaine, crack, crystal meth, and heroin using sex workers.
Sexton, 2019 [56]	United States	Cross-sectional	*n* = 273,720, 21% female, aged 12–17	Healthy	NA	NA	NSDUH (2008–2016)	Classic psychedelics defined as LSD, mescaline, DMT, psilocybin). Novel psychedelics defined as 5HT_2AR_ agonists with similar PK and PD to psychedelics. OR of lifetime novel psychedelic use vs. lifetime classic but not novel psychedelic use: Suicidal thinking: 1.4 (*p* = 0.0180) Suicidal planning: 1.6 (*p* = 0.0285) Past year suicide attempt: 1.2 (*p* = 0.6310) OR of lifetime novel psychedelic use vs. no novel or classic psychedelic use: Suicidal thinking: 1.3 (*p* = 0.0749) Suicidal planning: 1.4 (*p* = 0.1196) Past year suicide attempt: 0.9 (*p* = 0.8813)	Novel psychedelic use was associated with increased suicidal thinking and planning, relative to both classic and no psychedelic use.
Sexton, 2020 [57]	United States	Cross-sectional	*n* = 356,046, 51.5% female, aged 12–17	Healthy	NA	NA	NSDUH (2008–2017)	Lifetime classic tryptamine use vs. no use on past-year suicidal thinking: aOR = 0.79 Lifetime novel phenethylamine use vs. no use on past year suicidal thinking: aOR = 1.60 (No *p*-values reported)	While lifetime tryptamine use may decrease suicidal thinking, novel phenethylamine use is associated with increased suicidal thinking.
Zeifman, 2020 [58]	United States	Secondary analysis, two longitudinal studies	*n* = 104, 29.8% female, mean age of 29.28	Non-clinical, but not healthy	NA	Participants planned to use a psychedelic and responded to surveys before/after use.	Composite SI (QIDS-SI + SIDAS)	Psychedelics defined: psilocybin, LSD, ayahuasca, 5-MeO-DMT, Salvia divinorum, mescaline, ibogaine Composite score ranging from 1 to −1. Baseline: 0.48 2 weeks: −0.19 (*p* < 0.001; baseline to 2 weeks) 4 weeks: −0.47 (*p* < 0.003; 2 weeks to 4 weeks)	Intentional psychedelic use is associated with reductions in suicidality.
			*n* = 254, 45.3% female, mean age of 43.61	Non-clinical	NA	Participants planning to use psychedelics in the presence of a facilitator.	Composite SI (QIDS-SI + SIDAS)	Psychedelics defined: psilocybin, LSD, ayahuasca, 5-MeO-DMT, Salvia divinorum, mescaline, ibogaine, Composite score ranging from 1 to −1. Baseline: 0.16 4 weeks: −0.28 (*p* < 0.001 from baseline to 4 weeks)	Intentional psychedelic use in the presence of a facilitator is associated with reductions in suicidality.
Desai, 2022 [59]	United States	Cross-sectional	*n* = 10,504, 37.6% female, school-going adolescents	Healthy	NA	NA	YRBSS (2001–2019)	Psychedelics defined: LSD, PCP, mescaline, mushrooms OR for hallucinogen users vs. non users: Considered suicide 1.31 (*p* = 0.03) Made a suicide plan 1.44 (*p* = 0.03) Attempted suicide: 1.15 (*p* = 0.395) Injurious suicide attempt: 1.39 (*p* = 0.118)	Hallucinogen use is associated with increased suicidal ideation and attempt in school-going adolescents.
Jones, 2022 [60]	United States	Cross-sectional	*n* = 5983 psychedelic users, 41% female, mean of 16.08, aged 12–17	Healthy	NA	NA	NSDUH (2004–2019)	Psychedelics defined: LSD, psilocybin, peyote, mescaline aOR of Psychedelic use vs. no use on: (* = *p* ≤ 0.05) Lifetime suicidal thinking: MDMA: 0.96 Psilocybin: 0.84 * LSD: 1.19 * Peyote: 0.99 Mescaline: 0.80 Lifetime suicidal planning: MDMA: 1.12 Psilocybin: 0.78 * LSD: 1.36 * Peyote: 1.29 Mescaline: 0.72 Lifetime suicidal attempt: MDMA: 1.15 Psilocybin: 0.77 * LSD: 1.23 * Peyote: 1.24 Mescaline: 0.71	While psilocybin use was associated with reduced suicidal thoughts and behaviors, LSD use is associated with increased suicidal thoughts and behaviors. MDMA was not associated with suicidal thoughts and behaviors, and results for peyote and mescaline are nonsignificant as well.
Jones, 2022 [25]	United States	Cross-sectional	Total *n* = 484,732, aged 12–17 MDMA users *n* = 34,416, 42.2% female Psilocybin users *n* = 45,565, 33.5% female	Healthy	NA	NA	NSDUH (2008–2019)	OR of MDMA vs. no use on: Suicidal thinking: 0.9 (*p* = 0.01) Suicidal planning: 0.88 (*p* = 0.05) Suicide attempt: 1.00 (no *p*-value reported) OR of psilocybin vs. no use on: Suicidal thinking: 0.9 (*p* = 0.01) Suicidal planning: 0.88 (*p* = 0.08) Suicide attempt: 0.85 (*p* = 0.07) OR of LSD use vs. no use on suicidal thinking: 1.07 (*p* = 0.05)	MDMA and psilocybin are associated with decreased suicidal thinking and planning. Psilocybin is also associated with reduced suicide attempts. Unlike MDMA and psilocybin, LSD is associated with increased suicidal thinking.
Yang, 2022 [61]	United States	Cross-sectional	*n* = 241,675, aged 12–17	Healthy	NA	NA	NSDUH (2015–2020)	Tryptamines defined as DMT, AMT, Foxy aOR of LSD use vs. no use on Suicidal thinking: 1.21 (*p* < 0.001) Suicidal planning: 1.14 (*p* = ns) Suicidal attempt: 1.27 (*p* = ns) aOR of tryptamine (DMT/AMT/Foxy) use vs. no use on Suicidal thinking: 1.14 (*p* = ns) Suicidal planning: 1.81 (*p* < 0.01) Suicidal attempt: 1.16 (*p* = ns) aOR of Salvia divinorum vs. no use on Suicidal thinking: 1.41 (*p* < 0.05) Suicidal planning: 1.74 (*p* = ns) Suicidal attempt: 2.05 (*p* = ns) aOR of ecstasy use vs. no use on Suicidal thinking: 0.86 (*p* < 0.05) Suicidal planning: 0.80 (*p* = ns) Suicidal attempt: 0.83 (*p* = ns)	LSD use is associated with increased suicidal thinking, planning, and attempt. Salvia divinorum has an even greater association with such parameters than LSD, while ecstasy has a weaker association. Tryptamine effects on suicidal planning, thinking, and attempt are comparable to LSD, except that suicidal planning has a stronger association with tryptamine use.
Giugovaz, 2024 [62]	United States	Cross-sectional	*n* = from 25,800 to 42,800 per year, on average 53.6% female, aged 12–17	Healthy	NA	NA	NSDUH (2008–2020)	Psychedelics defined: LSD, MDMA, psilocybin OR for hallucinogen psilocybin users vs. non users: Suicidal ideation: 0.46 Suicidal planning: 0.56 Suicidal attempt: 1.01	LSD, MDMA, and psilocybin use is associated with reduced suicidal ideation and planning among adolescents.
Soboka, 2024 [63]	Canada	Cross-sectional	*n* = 22,500, 39% female, mean age of 37.09	Healthy	NA	NA	MHA intake (2020–2021)	aOR of psychedelic use vs. no use on Mild suicide risk: 2.04 (*p* < 0.001) Moderate/high suicide risk: 3.54 (*p* < 0.001)	Psychedelic use was moderately associated with mild suicide risk, and has an even stronger association with moderate/high suicide risk.
Relevant Items on Outcome Measures NSDUH: The NSDUH annually collects data via online and in-person interviews and includes questions on the following suicidal outcomes: ideation (i.e., at any time in the past 12 months, did you seriously think about trying to kill yourself?), planning (during the past 12 months, did you make any plans to kill yourself?), and attempt (i.e., in the past 12 months, did you try to kill yourself?). Answers to these questions are “yes”, “no”, “I don’t know”, and “I don’t want to answer”. YRBSS: Four dichotomized questions on the survey asked about the following suicide-related outcomes: considered suicide (during the past 12 months, did you ever seriously consider attempting suicide?), made a suicide plan (during the past 12 months, did you make a plan about how you would attempt suicide?), attempted suicide (during the past 12 months, how many times did you actually attempt suicide?), had an injurious suicide attempt (if you attempted suicide during the past 12 months, did any attempt result in an injury, poisoning, or overdose that had to be treated by a doctor or nurse?). MHA Intake: The following dichotomous suicide-related questions were asked: “Have you had thoughts about suicide or wanting to be dead in the past two weeks?” “Have you tried to kill yourself or attempt suicide in the past?” “Do you have thoughts of suicide now?” If participants had answered yes to any one of these questions, they were further interviewed and classified into a risk group by a clinician using their judgement and Nova Scotia’s suicide risk assessment and intervention tool (SRAI).									

Abbreviations: AE: adverse event. OR: odds ratio. aOR: odds ratio adjusted for covariates. PD: pharmacodynamics. PK: pharmacokinetics. Composite SI: Composite Suicidality Item. QIDS-SI: Quick Inventory of Depressive Symptomatology-Suicidality item. SIDAS: Suicidal Ideation Attributes Scale. NSDUH: National Survey on Drug Use and Health. YRBSS: Youth Risk Behavior Surveillance System. ESCAPAD: The Survey on Health and Drug Use on National Defence and Citizenship Day. MHA Intake: Mental Health and Addictions intake program of Nova Scotia. AESHA: An Evaluation of Sex Workers Health Access.

## Data Availability

Not applicable.

## Supplementary Materials

The following supporting information can be downloaded at https://www.mdpi.com/article/10.3390/jcm14051416/s1.
