# Supplementary material for: Psychedelics and Suicide-Related Outcomes: A Systematic Review
Source: J Clin Med. 2025 Feb 20;14(5):1416. doi: 10.3390/jcm14051416 (PMC11900607; doi:10.3390/jcm14051416)
Supplement: Supplementary file 1 [file jcm-14-01416-s001.zip › jcm-3435885-supplementary.pdf]

Supplementary Materials
Table S1. Quality assessment of included published studies.

**Observation Study /uncontrolled/ open-label Study Questions - NOS**	Aaronson, 2023	Carhart-Harris et al., 2018	Anderson et al., 2020	Ellis, 2024	Carbonaro, 2016	Celine, 2019	Kim, 2011	Davis, 2023	Davis, 2020	Ragnhildstveit, 2023	Han, 2022	Giugovaz, 2024	Desai, 2022	**Argento, 2017**	Argento, 2018	Sexton, 2019	Sexton, 2020	Hendricks, 2015	Jones, 2022	Jones, 2022	Wong, 2013	Yang, 2022	Soboka, 2024
Representativeness of the exposed cohort																							
Selection of the non-exposed cohort																							
Ascertainment of exposure																							
Demonstration that outcome of interest was not present at start																							

of study																							
Comparability of cohorts on the basis of the design or analysis is controlled for confounders																							
Assessment of outcome																							
Was follow-up long enough for outcomes to occur																							
Indicate the median duration of follow-up and a brief rationale for the assessment above																							
*Poor because poor comparability score																							

**RCT Study Questions - Cochrane**	Davis et al., 2021	Gasser et al., 2015	Carhartt-Harris et al., 2021	Goodwin et al., 2022	Peck et al., 2022	Ross et al, 2021	Von Rotz et al., 2022	Olatora et al., 2018	Raison et al., 2023	Mitchell et al, 2021	Mitchell et al. 2023	Mithoefer et al., 2018	Mithoefer et al, 2018
Random sequence generation	Low risk	Low risk	Low risk	Low risk	Low risk	Low risk	Low risk	Low risk	Low risk	Low risk	Low risk	Low risk	Low risk
Allocation concealment	Some concerns	Low risk	Low risk	Low risk	Some concerns	Low risk	Low risk	Low risk	Low risk	Low risk	Low risk	Low risk	Low risk
Blinding of participants and personnel	Low risk	Low risk	Low risk	Low risk	Low risk	Low risk	Low risk	Low risk	Low risk	Low risk	Low risk	Low risk	Low risk
Blinding of outcome assessment	Low risk	Low risk	Low risk	Low risk	Low risk	Low risk	Low risk	Low risk	Low risk	Low risk	Low risk	Low risk	Low risk
Incomplete outcome data	Low risk	Low risk	Low risk	Low risk	Low risk	Low risk	Low risk	Low risk	Low risk	Low risk	Low risk	Low risk	Low risk
Selective reporting	Low risk	Low risk	Low risk	Low risk	Low risk	Low risk	Low risk	Low risk	Low risk	Low risk	Low risk	Low risk	Low risk
